# Transcriptional Analysis of the Effects of Gambogic Acid and Neogambogic Acid on Methicillin-Resistant *Staphylococcus aureus*

**DOI:** 10.3389/fphar.2019.00986

**Published:** 2019-09-13

**Authors:** Xin Hua, Yue Jia, Qin Yang, Wanjiang Zhang, Zhimin Dong, Siguo Liu

**Affiliations:** ^1^Division of Bacterial Diseases, State Key Laboratory of Veterinary Biotechnology, Harbin Veterinary Research Institute, Chinese Academy of Agricultural Sciences, Harbin, China; ^2^College of Life Science, Northeast Forestry University, Harbin, China; ^3^Innovation Team of Livestock and Poultry Epidemic Disease Prevention and Control, Tianjin Animal Science and Veterinary Research Institute, Tianjin, China

**Keywords:** MRSA, *staphylococcus aureus* biofilms, saeRS two-component system, gambogic acid, neogambogic acid

## Abstract

Methicillin-resistant *Staphylococcus aureus* (MRSA) infection is a major threat to human health, as this bacterium has developed resistance to a variety of conventional antibiotics. This is especially true of MRSA biofilms, which not only exhibit enhanced pathogenicity but also are resistant to most antibiotics. In this work, we demonstrated that two natural products with antitumor activity, namely, gambogic acid (GA) and neogambogic acid (NGA), have significant inhibitory activity toward MRSA. GA and NGA can not only effectively inhibit planktonic MRSA strains *in vivo* and *in vitro*, but also have strong inhibitory effects on MRSA biofilms formation. By transcriptome sequencing, Q-RT-PCR and PRM, we found that GA and NGA could reduce the expression of *S. aureus* virulence factors by inhibiting the *saeRS* two-component, thus achieving inhibition of MRSA. We found that GA and NGA had anti-MRSA activity *in vivo* and *in vitro* and identified *saeRS* to be the target, indicating that *saeRS* inhibitors may be used to treat biofilm-related infections.

## Introduction

Methicillin-resistant *Staphylococcus aureus* (MRSA) can induce multiple human diseases, such as necrotic pneumonia, endocarditis, and septicemia (Tenover and Goering, 2009; Alam et al., 2015; David and Daum, 2017). In the United States, it is estimated that the mortality rate due to MRSA infection is higher than that of HIV/AIDS and tuberculosis (Infectious Diseases Society of America et al., 2011); nearly 11,000 people die each year from MRSA infection (Mohammad et al., 2015; Thangamani et al., 2015b). MRSA is considered to be a major public health concern in hospital and community settings (Tavares et al., 2014; Lehar et al., 2015; Udo and Al-Sweih, 2017). Due to significant resistance of MRSA to a wide range of antibiotics, treatment tends to be ineffective, especially after biofilm formation, which limits the number of therapeutic options available (Pozzi et al., 2012; Ohadian Moghadam et al., 2014; Vazquez-Sanchez et al., 2018). MRSA is a challenge for the medical field worldwide, and antibiotics remains the major method of treatment. Regrettably, it takes a long time to develop new antibiotics, and antibiotic development has not been able to keep pace with the emergence of new generations of resistant bacteria. Hence, the development of novel therapeutic agents and antibiotic substitutes with activity against highly pathogenic bacteria is urgently required.

The high pathogenicity and mortality rate due to *S. aureus* infection are mainly attributed to the various virulence factors produced by this bacterium (Ferro et al., 2016). These secreted toxins are associated with host tissue infection, immune evasion and bacterial pathogenesis (Miyazaki et al., 2012; Den Reijer et al., 2016; Ferro et al., 2016). MRSA toxins and biofilms directly affect wound healing in patients, leading to further systemic complications (Smith et al., 2010; Federman et al., 2016). In *S. aureus*, the expression of these virulence factors is controlled by a network of transcription factors (such as *mgrA*, *sarA*, *sigB,* and *rot*) and two-component regulatory systems (such as *srrAB*, *arlRS*, *vraSR*, and *saeRS*) (Boyle-Vavra et al., 2006; Cho et al., 2015; Guo et al., 2017). As a major signal transduction mechanism in bacteria, two-component signaling (TCS) is responsible for adaptation to environmental changes *via* the sensing of various cues (such as nutrient concentration, ionic strength, and membrane interference) (Giraudo et al., 1999; Fournier and Hooper, 2000; Hall et al., 2017). The *saeRS* two-component system plays a vital role in the expression and pathogenesis of *Staphylococcus* virulence genes and can regulate more than 20 virulence factors, such as coagulase, alpha-hemolysin and fibronectin-binding proteins. Although the *saeRS* two-component system has been reported to be directly associated with the formation of *S. aureus* biofilms, drugs targeting *saeRS* have not been developed (Cho et al., 2015; Liu et al., 2016; Guo et al., 2017).

GA and NGA are two active compounds found in *Garcinia* species, which exhibit immune-enhancing, anti-inflammatory, antitumor, and proapoptotic activities (Wang et al., 2011; Chen et al., 2015; Zhang et al., 2016; Jin et al., 2018). Especially in the aspects of anti-inflammatory and anti-tumor, it has been found that gamoic acid can inhibit many cell signaling pathways, such as nuclear factor-kappa B (nf-κb), tumor necrosis factor-α (TNF-α), and iNOS (Pandey et al., 2016; Sun et al., 2018). It has been reported that a series of xanthone derivatives, including GA, have anti-MRSA strain activity, and could disrupt intracellular invasion of *S. aureus*, but no further research has been conducted on the mechanism of action (Chaiyakunvat et al., 2016). In addition, there are few reports on the antibacterial activities of GA or NGA.

In this paper, we demonstrated the inhibition of MRSA and the activity against biofilm formation by GA and NGA *in vivo* and *in vitro*. This antibacterial activity is mainly achieved by inhibiting the expression of multiple virulence factors in MRSA, which in turn occurs *via* inhibition of the *saeRS* two-component system. In this study, we reported for the first time that GA and NGA have the activity of inhibiting MRSA biofilm formation, and revealed the new mechanism of the antimicrobial activity of GA and NGA. This study provides favorable evidence for the study of the anti-bacterial mechanism of GA and NGA.

## Materials and Methods

### Strains and Growth Conditions

Clinical MRSA and MSSA isolates were kindly donated by the First Affiliated Hospital of Harbin Medical University, Harbin, China. The *S. aureus* standard strains ATCC29213 (methicillin-sensitive *staphylococcus aureus*) and ATCC 33591 (MRSA) (American Type Culture Collection, USA) and the clinical MRSA strain maintained in Mueller-Hinton broth (MHB, Oxoid, Basingstoke, England) were frozen at −80°C before use. Details regarding the strains have been provided in previous reports (Hua et al., 2018).

### Antimicrobial Agents

**Figure 1:**
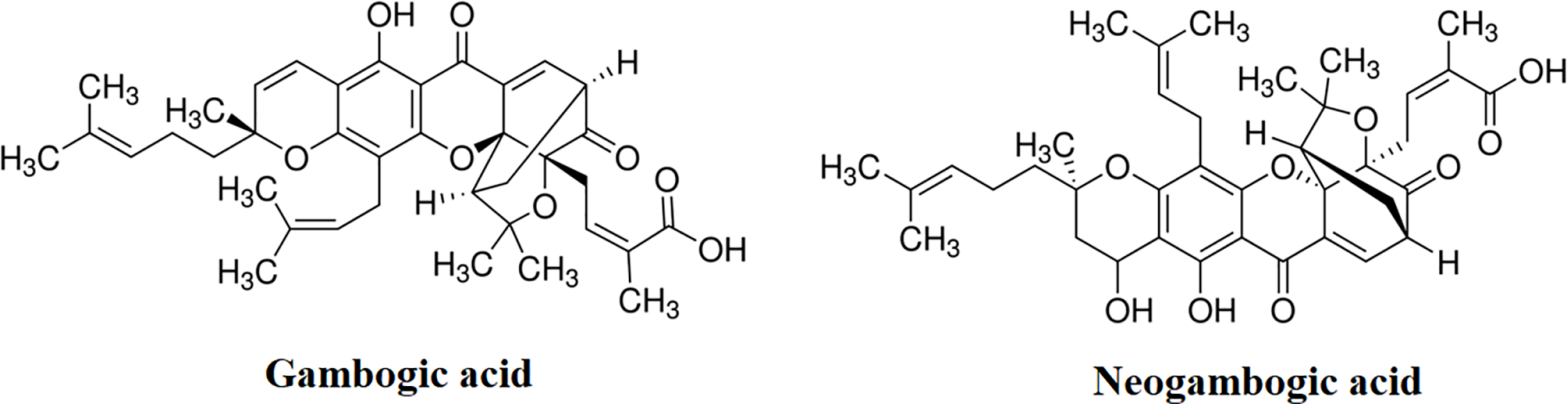
Chemical structure of gambogic acid and neogambogic acid. (https://www.sigmaaldrich.com/catalog/product/sigma/g8171?lang=zh&region=CN; https://www.sigmaaldrich.com/catalog/product/supelco/phl83527?lang=zh&region=CN)

GA, NGA, vancomycin, and linezolid were purchased from Sigma Aldrich (Bornem, Belgium), the structural formula of GA and NGA are shown in **Figure 1**. GA and NGA were dissolved in DMSO. Vancomycin and linezolid were dissolved in ultrapure water.

### MIC and MBC Measurement

Based on the CLSI guidelines, broth micro dilution was adopted to determine the MIC and MBC values. Briefly, for MIC and MBC determination, the test medium was Trypticase soy broth (TSB) and the density of bacteria was 5×10^5^ colony forming units (CFU)/mL. Cell suspensions (200 μL) were inoculated into the wells with antibiotics at different final concentrations (32, 16, 8, 4, 2, 1, 0.5, and 0.25 mg/mL). The inoculated microplates were incubated at 37°C for 16 h before being read. The MIC and MBC were interpreted as the lowest concentration of antibiotic that completely inhibited the visible growth or killed the bacteria. The experiment was carried out in triplicate.

### Cytotoxicity Assay

HeLa cells and mouse skin keratinocytes cells (CP-M168, form Procell Life Science&Technology Co., Ltd. Wuhan, China) were seeded at a density of 10,000 cells per well in a 96-well cell culture plate (NEST, Nest Biotech Co., Ltd., NJ, USA) and incubated overnight at 37°C in dulbecco’s modified eagle medium (DMEM) containing 10% fetal bovine serum (FBS). Then, the cells were treated with GA and NGA for 24 h at different concentrations from 0 to 128 μg/mL. The treated cells were washed four times with PBS, and DMEM containing MTS (20%) assay reagent (3-(4,5-dimethylthiazol-2-yl)-5-(3carboxymethoxyphenyl)-2-(4-sulfophenyl)-2H-tetrazolium; Promega, Madison, WI, USA) was added. After 4 h of incubation at 37°C, the absorbance was measured using an ELISA microplate reader (Molecular Devices, Sunnyvale, CA, USA). The percent cell viability of the GA- and NGA-treated cells was calculated.

$$\mathrm{cell viability}100\%=\frac{0\mathrm{Dtreated group}}{0\mathrm{Dcontrol group}}$$

### Time-Dependent Killing

An overnight cell culture (*S. aureus* ATCC33591, about 1×10^10^) was diluted 1:5,000 in MHB and incubated at 37°C and 220 rpm for 2 h. Then, the bacterial cells were treated with GA, NGA or vancomycin at a concentration of 5 × MIC. One milliliter of each culture was removed at specific time intervals and centrifuged at 10,000 × g for 1 min. The pellet was resuspended in 100 mL of sterile PBS. Diluted suspensions were plated on Mueller-Hinton agar (MHA) and incubated at 37°C overnight for CFU calculation. Experiments were performed with three replicates.

### Virulence Factor Detection

The effects of GA, NGA, linezolid, and vancomycin on the production of two important *S. aureus* toxins (Hla and Panton-Valentine leukocidin (PVL)) was measured by utilizing ELISA as described previously (Thangamani et al., 2015b).

### Inhibition of Adhesion and Infection

The adhesion and infection experiments were performed as described previously with some modifications (Frandoloso et al., 2012; De Llano et al., 2015). In brief, MRSA ATCC35391 was exposed to GA and NGA at a concentration of 0.25 µg/mL and cultured at 37°C until the cell growth reached the logarithmic phase and a bacterial cell density of 1×10^9^ CFU/mL was achieved. A prepared monolayer of keratinocytes cells (1×10^6^ cells/pore) was washed with phosphate buffer saline (PBS) to eliminate antibiotics and then covered with 1 mL of GA- and NGA-treated MRSA strains. The cells were cultured at 37°C in 5% CO_2_, and the unbound bacteria were removed by washing five times with PBS. One hundred fifty microliters of trypsin was added to digest and separate the cells from the adherent bacteria. In addition, 850 µL of deionized water was added and bubbled repeatedly to release the cells and the cell-associated bacteria. One hundred microliters of diluted lysis buffer was coated onto the MHA plate; the cells were cultured for 20 h; and the total number of CFUs was determined.

After the adhesion test, the unbound bacteria were removed by washing with PBS 5 times, and the infection test was conducted by adding DMEM containing gentamycin and incubating for 2 h to remove surface bacteria. The remaining procedure was the same as that for the adhesion test.

### Scanning Electron Microscope

Biofilm formation was conducted as described above with glass coverslips in 24-well plates. The biofilms formed were fixed with 2.5% glutaraldehyde in 0.1 M sodium cacodylate buffer (pH 7.2) at 4°C for 10 min and then washed with PBS three times. The biofilms were then fixed with 1% osmic acid at room temperature for 10 min. Then, gradual dehydration was carried out with ethyl alcohol (60, 70, 80, 90, 95, and 100%), and tertiary butanol was used as a displacement liquid (60, 70, 80, 90, 95, and 100%). Finally, the samples were freeze-dried overnight. The specimens were then sputter coated with gold for observation using a JSM 7500 (JEOL, Tokyo, Japan).

### Biofilm Assay

*S. aureus* ATCC 33591 was cultured in tryptic soy broth containing 1% glucose, and biofilms were formed after 24 h of incubation at 37°C. Then, the medium was removed, and the biofilms were washed with PBS. Drugs were added at concentrations of 0.25, 0.5, 1, and 2 µg/mL, and the biofilms were incubated for an additional 24 h at 37°C. The 96-well plate was washed again, and the biofilms were stained with 0.1% (wt/vol) crystal violet. Then, the 96-well plates were washed and air-dried, and finally, the biofilm mass was dissolved in 95% ethanol. A microplate reader (Bio-Tek Instruments Inc.) was used to measure the absorbance (490 nm) of the crystal violet. The data are presented as the percent biofilm mass reduction in the treated groups compared with that in the control group.

### Mouse Experiments

Eight-week-old female BALB/c mice (Vital River, Beijing, China) were used in all the mouse experiments. The animal experiments were performed in accordance with animal ethics guidelines and approved protocols. The animal experiments were approved by the Animal Ethics Committee of the Harbin Veterinary Research Institute of the Chinese Academy of Agricultural Sciences (approval number IACUC-2018-086).

In systemic nonlethal infection, mice were intraperitoneally injected with 1.2×10^8^ CFUs of *S. aureus* ATCC33591. The mice were then divided into four groups (15 mice per group) and tail vein injected with GA (5 mg/kg), NGA (5 mg/kg), vancomycin (5 mg/kg) or vehicle (10% ethanol) alone. The mice were treated once daily for six days and euthanized after 24 h of the final administration. Organs (including heart, lung, kidney, spleen, and liver) were excised for histological analyses. Mice in the control and treated groups were subjected to the same systemic nonlethal infection protocol and submitted for histopathological examination after six days.

Skin infections were performed according to the infection model used by Purdue University with slight modification (Thangamani et al., 2015a). Brieﬂy, mice (10 mice in each group) were injected intradermally with 4.5×10^8^ CFUs of MRSA ATCC33591, and after 48 h, formation of an open wound was observed at the injection site. Then, the mice were treated with 1% GA or 1% NGA (using 20 mg of petroleum jelly as the vehicle) once a day for 9 days; the control group was treated with the vehicle alone. On the fifth day, 5 mice were selected randomly; the area around the wound was lightly swabbed with 70% ethanol; and the wound (1 cm^2^) was excised, homogenized, serially diluted, and plated on MHA. The plates were incubated at 37°C for 18 h before counting the viable bacterial CFU. The remaining 5 mice in each group continued to be treated until day 9 to observe the effect of treatment on the wound.

### RNA-Seq Transcriptomics

*S. aureus* ATCC33591 was grown to an OD600 of 0.4 from an initial value of 0.01, and GA and NGA were added to a final concentration of 1/2 × MIC. Samples were collected 1 h post treatment and preserved with RNAprotect (Qiagen, USA) following the manufacturer’s instructions. The cells were pelleted by centrifugation at 5,000 × g for 10 min at 4°C. RNA was isolated using the RNeasy Mini Kit (Qiagen, USA) in accordance with the manufacturer’s instructions with the following modifications: The cell pellets were homogenized in 1 mL of Tris-buffered saline (TBS) (20 mM Tris, pH 7.5) containing 0.4 mg of lysostaphin and incubated at 37°C for 15 min. Subsequently, 20 mg of lysozyme in TE buffer (20 mM Tris, pH 7.5; and 2 mM ethylene diamine tetraacetic acid, pH 7.8) was added, and the sample was incubated at 25°C for 10 min. Control samples were collected from an antibiotic-free culture, and each experiment was repeated three times.

Three independently prepared RNA samples from each strain were used for RNA-Seq. Illumina sequencing was performed by Shanghai Majorbio Biopharm Technology Co., Ltd. (Shanghai, China) using the Illumina HiSeq2000 Truseq SBS Kit v3-HS (200 cycles) and the MiSeq Reagent Kit V2 (500 cycles/600 cycles) (Illumina Inc.). Data analyses were performed using edgeR software. Genes exhibiting 2-fold changes in expression, which were statistically significant as determined by Student’s t-test (p < 0.05), were considered to be differentially expressed under the conditions indicated.

### Real-Time RT-PCR

To verify the RNA-Seq data, we selected some genes that were downregulated and assessed the relative expression levels of these genes by real-time RT-PCR. *S. aureus* ATCC33591 cells were cultured under the same conditions as those of the RNA-Seq transcriptomics experiments. Q-RT-PCR was performed by a two-step process. These reactions were performed using an Applied Biosystems qTOWER 2.2 (Analytik Jena, Jena, Germany) real-time PCR system by using the following cycling parameters: 95°C for 5 min; 40 cycles of 95°C for 15 s, 55°C (for the cap5C gene) or 57°C for other genes for 15 s, and 72°C for 15 s; and one dissociation step of 95°C for 1 min, 55°C for 30 s, and 95°C for 30 s. All the measurements were independently conducted 3 times for 2 separate biological isolates. The sequences of all the primers used are listed in **Supplementary Table S1**.

A melting curve analysis was performed immediately after amplification to verify the specificity of the PCR amplification products. Fluorescence was measured at the end of the annealing-extension phase of each cycle. The threshold value for the fluorescence of all the samples was set manually. The reaction cycle at which the PCR product exceeded this fluorescence threshold was identified as the threshold cycle. Relative quantitation was performed by the 2−ΔΔCT method.

### Parallel Reaction Monitoring

Parallel reaction monitoring (PRM)-MS was performed by Shanghai Meiji Biology Co., Ltd. The expression levels of the proteins encoded by specific genes identified by RNA-Seq analysis were determined by quantifying the changes in the expression levels of the selected proteins before and after treatment with NGA. Specific peptide sequences were selected based on the proteins selected for PRM analysis. The chromatographic column used was a C18 column (75 μm × 25 cm; Thermo, USA) liquid chromatography was performed on an EASY-nLC 1200; the mass spectrometer used was a Q-Exactive Thermo, USA; the data acquisition software used was Thermo Xcalibur 4.0 (Thermo, USA); and Skyline software was used for quantitative analysis of the proteomics data.

### Statistical Analysis

Statistical analyses were performed using GraphPad Prism 6.0 (GraphPad Software, La Jolla, CA). One-way ANOVA was performed between groups. For ANOVA, the observed variance is partitioned into components according to different explanatory variables. *P < 0.05 was considered to be significant.

## Results and Discussion

### Inhibitory Activity of GA and NGA Toward MRSA

To assess the antibacterial activity of GA and NGA, 20 strains of MSSA and MRSA were selected. According to the MIC results (**Table 1**), both GA and NGA exhibited excellent inhibitory activity toward MRSA and MSSA. The MIC values for the inhibition of MSSA ranged from 0.5 μg/mL to 4 μg/mL. For MRSA inhibition, although the MIC of oxacillin was 64 μg/mL, the MICs of GA and NGA remained between 0.5 μg/mL and 4 μg/mL. Earlier reports show that the MIC of GA on MRSA strains USA3000 is 12.5 μM (Chaiyakunvat et al., 2016). In this study, we used the ATCC33591 strains and the rest of the 19 clinical strains. While the MIC of GA and NGA on all strains were between 0.5 and 4 μg/mL, significantly lower than that reported. Bacterial killing curve and in vivo experiment results also show that the GA and NGA have very strong antibacterial activity (Chaiyakunvat et al., 2016).

**Table 1:** MIC of GA and NGA against *Staphylococcus aureus* strains.

	MSSA					MRSA			
	Strains	GA		NGA	Oxacillin	Strains	GA	NGA	Oxacillin
	MIC (μg/mL)					MIC (μg/mL)			
Standard strain	ATCC29213		1	1	0.25	ATCC33591	1	1	128
Clinical isolates	L1		0.5	1	0.25	LN2	0.5	1	>128
	L2		0. 5	2	0.5	LN3	0.5	0.5	>128
	L4		1	2	0.25	LN4	1	0.5	64
	L5		1	1	0.25	LN6	1	1	>128
	L7		0.5	1	1	LN8	2	2	>128
	L11		4	4	0.25	LN18	1	0.5	>128
	L13		2	1	0.25	LN19	4	4	64
	L17		1	1	0.25	LN20	1	1	>128
	L22		1	0.5	0.25	LN21	2	4	>128
	L23		1	0.5	0.25	LN22	2	2	>128
	L24		1	2	1	LN23	1	1	128
	L28		2	2	0.25	LN30	2	2	>128
	L30		1	1	0.5	LN33	1	1	>128
	L31		1	1	0.25	LN36	1	1	>128
	L32		0.5	0.5	0.25	LN44	2	1	128
	L37		4	2	0.25	LN45	1	0.5	>128
	L40		1	2	0.5	LN46	1	1	>128
	L55		0.5	1	0.25	LN50	0.5	1	>128
	L56		1	0.5	0.25	LN58	0.5	0.5	>128
	L57		1	0.5	0.25	LN63	1	1	>128

### Cytotoxicity

GA and NGA are extracted from the traditional Chinese medicine gamboge. It has been reported that the IC_50_ of GA on toxicity standard cell line L929 cells was 287 μg/mL, and acute injection toxicity indicated that the half lethal dose (LD_50_) of GA was (18.59 mg/kg, 95% LD_50_, 16.84–20.53 mg/kg) (Feng et al., 2018). GA had no significant side e?ects on cardiovascular, respiratory, and central nervous systems at higher doses (16 μg/kg)(Zhao et al., 2010). Although there have been many reports of strong inhibitory effects of GA and NGA on a variety of tumor cells (Pandey et al., 2016; Sun et al., 2018), while GA and NGA have also been shown to be safe for normal cells and humans.

### Kinetics of Bacterial Killing

**Figure 2:**
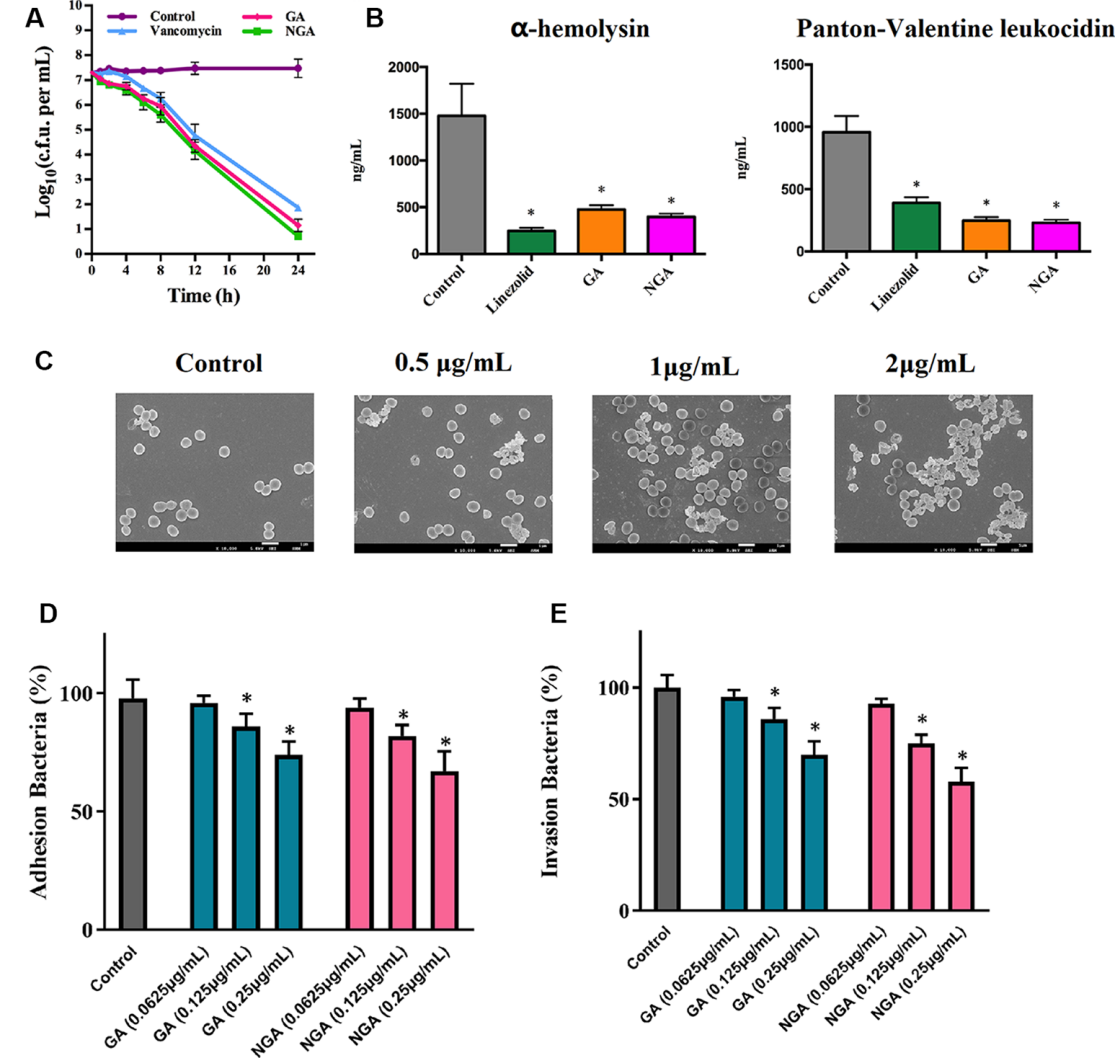
*In vitro* antibacterial activity of gambogic acid and neogambogic acid. **(A)** Time-kill kinetics of GA and NGA against *S. aureus* ATCC33591. **(B)** Toxin production (ng/mL) in *S. aureus* ATCC33591 after treatment with GA (1μg/mL ), NGA (1μg/mL) or linezolid (8μg/mL) for one hour. The results are presented as mean ± SD (n = 3). **(C)** Scanning electron microscopy images showing the structure of *S. aureus* ATCC33591 treatment with GA for one hour. Magnifications, x 10,000. **(D)** and **(E)** The adherence and invasion of *S. aureus* ATCC33591 with/without GA and NGA treatment to keratinocytes cells CM-M168. Statistical analysis was done by One-way ANOVA test between groups. P values of (*P ≤ 0.05) are considered significant.

The rates of microbial killing by GA, NGA, and vancomycin were determined by exposing MRSA ATCC33591 cells to 5×MIC of each treatment over a 24-hour incubation period at 37°C. Both GA and NGA exhibited a rapid bactericidal effect, with a 3-log10 reduction (99.9% clearance) within 4 and 6 h, respectively (**Figure 2A**). In comparison, vancomycin achieved a 3-log10 bacterial reduction only after 24 h.

Compared to the control group, the GA and NGA treatment groups exhibited significant suppression of two key toxins (PVL and Hla, that injure host immune cells and promote infection of host tissues) by MRSA ATCC33591. GA and NGA exhibited better inhibitory activity toward PVL and Hla than linezolid (an antibiotic that inhibits protein synthesis) (**Figure 2B**).

Scanning electron microscopy (SEM) was utilized to observe the surface morphology of ATCC33591 before and after GA and NGA treatment. The cell walls of ATCC33591 exhibited contraction and rupture after treatment with GA and NGA, and this condition worsened with increasing concentration (**Figure 2C**). When the concentration of GA and NGA was at 2 × MIC, most of the bacteria died.

### Inhibition of Adhesion and Invasion

The inhibitory effects of GA and NGA toward ATCC33591 cells adhered to keratinocytes cell were as shown in **Figure 2D**. With increasing concentration of GA and NGA, inhibition of MRSA infection increased gradually. When the concentration of GA and NGA reached 0.25 µg/mL, the adhesion rate were 69.9% and 57.6%, respectively, compared with the control group, and this difference was statistically significant (P < 0.05). Similar to the results observed for adhesion, the infection ability of the ATCC33591 strain treated with GA and NGA also decreased in a concentration-dependent manner (**Figure 2E**). When the concentration of GA and NGA was 0.25 µg/mL, the rates of invasion were 73.5% and 66.6% compared with the control group, respectively.

### Inhibition of Biofilm Formation

Staphylococcal biofilms are intrinsically resistant to conventional antibiotics, and currently, there are no effective therapies that target microbial biofilms. Therefore, novel antibiofilm agents, treatments and strategies are needed. Since GA and NGA exhibited significant activity against planktonic bacteria, the inhibition of biofilm formation was tested.

**Figure 3:**
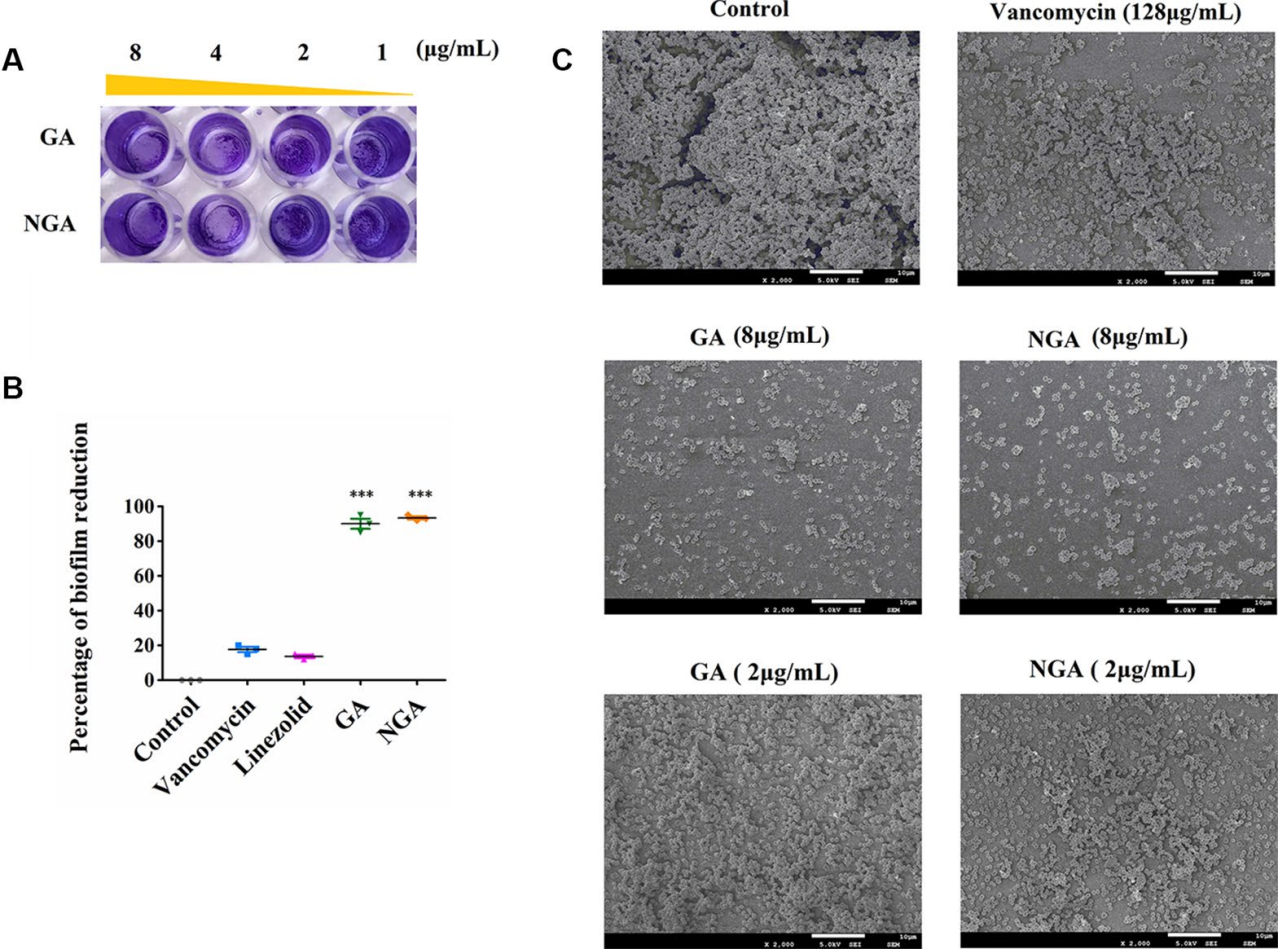
Gambogic acid and neogambogic acid inhibit MRSA biofilm formation *in vitro*. **(A)** Crystal violet assay to assess the antibioflm activity of GA and NGA against *S. aureus* ATCC33591 bioflm. **(B)** Percent reduction of *S. aureus* ATCC33591 biofilm after treatment with GA and NGA (8μg/mL). **(C)** Scanning electron microscopy images showing the structure of *S. aureus* ATCC33591 biofilm. Magnifications, x 2,000. P values of (***P ≤ 0.005) are considered significant.

The *in vitro* effects of GA and NGA on MRSA biofilm formation were investigated using semiquantitative crystal violet staining assays and SEM. As shown in **Figures 3A, B**, GA and NGA could significantly inhibit the growth of biofilms at 2 μg/mL in the crystal violet experiment, and the inhibitory effect became more apparent as the drug concentration increased. Eighty-seven percent of the biofilm formation was inhibited by GA and NGA at 8 μg/mL, and similar results were observed by SEM (**Figure 3C**). The biofilms were observed to be thick by SEM; however, after treatment for 4 h with 8 μg/mL NGA or 8 μg/mL GA, the bacterial abundance was greatly reduced, and the bacteria failed to form biofilm structures.

### *In Vivo* Experiments

**Figure 4:**
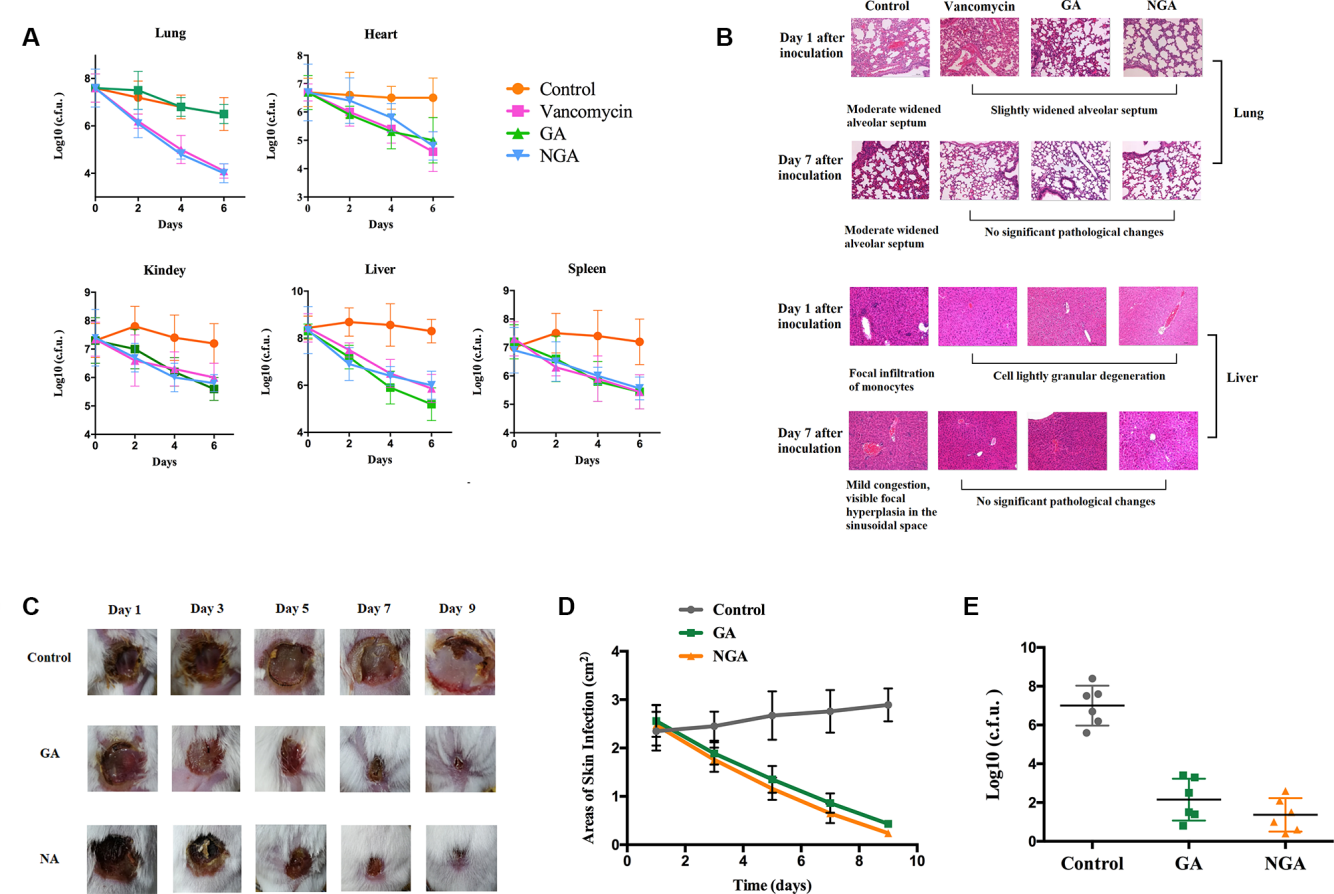
GA and NGA can effectively inhibit septicemic and skin infection caused by *S. aureus* ATCC33591 *in vivo*. **(A)** Fifteen mice per group were infected (i.p) with non-lethal dose of *S. aureus* ATCC33591 and treated orally with GA, NGA, vancomycin (5 mg/kg) or the vehicle alone for six days (one dose per day). 24 h after the last treatment, mice were euthanized and their organs were excised and homogenized in TSB to count viable MRSA colonies. The number of CFU from each mouse is plotted as individual points. Values are the mean of triplicate results with standard deviation bars. **(B)** Histological evaluation of lung and liver of mice infected with *S. aureus* ATCC33591 receiving no treatment or a treatment with GA and NGA. Both lung and liver in control group demonstrated acute inflammation, in the treated, group no apparent pathological changes were observed. **(C)** Ten mice per group with subcutaneous infection *S. aureus* ATCC33591. After the wound is formed the mice were treated with 1% GA or 1% NGA once a day for 9 d. Compared with the control group, the wounds healed well after GA and NGA treatment, the wound area **(D)** and the amount of bacteria **(E)** were significantly reduced.

A mouse sepsis model was used to evaluate the antibacterial activity of GA and NGA *in vivo*. Mice were intraperitoneally injected with 1.2×10^8^ CFUs of ATCC33591 and provided 5 mg/kg GA or NGA daily. As depicted in **Figure 4A**, treatment with GA, NGA and vancomycin led to significant reduction in the mean bacterial load in different organs. In particular, both treatments reduced the mean bacterial load by more than 1000-fold in the lungs. The histopathological inspection performed six days after infection with a nonlethal dose of MRSA ATCC 33951 revealed no changes in the heart, spleen and kidneys. While the animals exhibited moderate histopathological alterations in the lungs and liver in the control group, after treatment with GA, NGA, or vancomycin, there were no obvious histopathological alterations (**Figure 4B**).

We monitored skin necrosis in mice on days 1, 3, 5, 7 and 9 after infection with MRSA. As shown in **Figures 4C, D**, the areas of skin infection on the mice decreased significantly after treatment with GA and NGA, and the infection also decreased significantly. Mouse skin was collected on day 5 after inoculation, and CFU enumeration was performed; there was a clear decrease in the amount of bacteria in the GA- and NGA-treated groups (**Figure 4E**).

### Transcriptomics

To determine how GA and NGA inhibit MRSA, transcriptomic studies were conducted. We compared the transcriptome of untreated ATCC33591 with those of the strain treated with GA and NGA. A total of 2,944 genes were detected; 149 and 178 genes were differentially expressewd in the GA- and NGA-treated groups compared with the control group, with 74 and 102 downregulated genes, respectively, and 75 and 76 upregulated genes, respectively. These differentially expressed genes were selected based on logFC values greater than 2 and p < 0.05. The GA and NGA data were the results of the interaction of each of these compounds with ATCC33591 and were similar because of the similar structures of GA and NGA. Except for the slight numerical difference between the two sets of data, most of the genetic change trends were consistent, which suggested the accuracy and reliability of the data.

**Figure 5:**
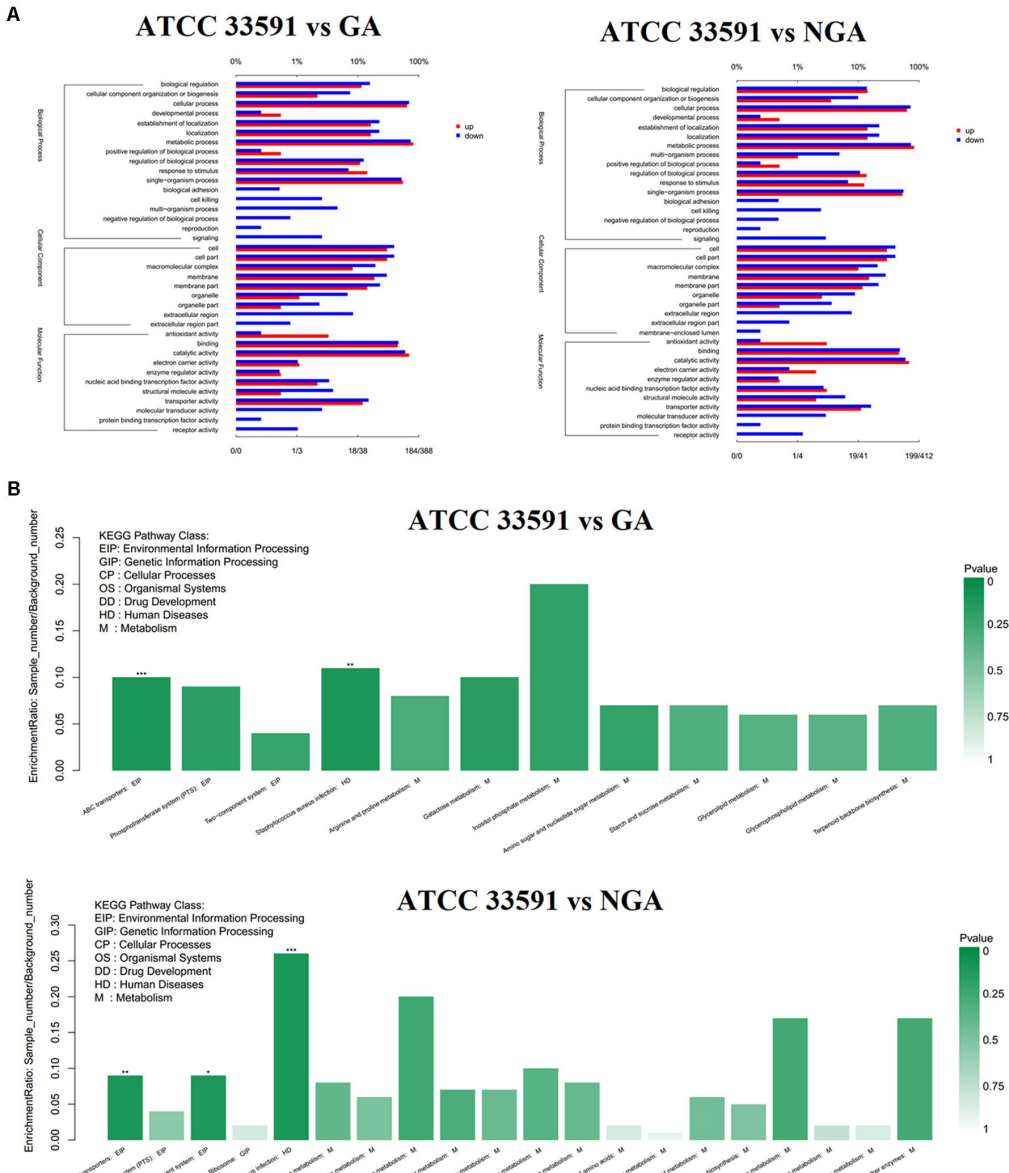
RNA-Seq gene expression results for *S. aureus* ATCC33591 cells treated and not treated with GA and NGA. **(A)** GO enrichment analysis of differently expressed genes. **(B)** Down-regulated genes enriched in the KEGG Pathway.

To better understand the functions of these differentially expressed genes, we conducted GO and KEGG distribution analyses. The differentially expressed genes were divided into three GO categories (**Figure 5A**) – cellular component, biological process, and molecular function – according to sequence homology. GO categories shows that the gene expression trends of the MRSA strains were quite similar after GA and NGA treatment. In terms of functional classification, genes associated with biological adhesion, cell killing, multiorganism process, negative regulation of biological process, and reproduction were significantly downregulated, while both biological adhesion and cell killing were key factors associated with biofilm formation. This result is consistent with our previous observations. In terms of cellular composition, the downregulated genes were mainly distributed in the extracellular region, and the downregulation was caused by the inhibition of some related virulence factors. Analysis of the molecular function showed that some activities were inhibited, such as signal transduction activity, protein-binding transcription factor activity, and receptor activity. The down regulated KEGG pathway analysis showed that the differentially expressed genes were mainly clustered in the ABC transporters, *Staphylococcus aureus* infection, and two-component system categories (**Figure 5B**).

Simultaneously, we further analyzed 102 downregulated genes. We found that there was significant downregulation of many genes associated with virulence factors, two-component regulatory systems, cell wall synthesis and several energy metabolism related genes, which was shown in **Table 2**.

**Table 2:** Key genes of ATCC33591 down-regulated by GA and NGA.

Gene ID	Gene name	Description	Log2 flod change (GA)	Log2 flod change (NGA)
SAV0023	None	5’-nucleotidase	–2.13	–2.16
SAV0095	plc	1-phosphatidylinositol phosphodiesterase	–2.67	–3.49
SAV0104	None	Na/Pi cotransporter	–2.28	–2.38
SAV0191	None	N-acetylmuramic acid-6-phosphate etherase	–2.9	–3.42
SAV0192	None	PTS system EIIBC component	–2.5	–2.82
SAV0193	None	RpiR family transcriptional regulator	–2.25	–2.56
SAV0216	None	arabinogalactan ABC transporter permease	–1.91	–2.17
SAV0217	None	oxidoreductase	–1.86	–2.39
SAV0218	None	NADH-dependent dehydrogenase	–1.86	–2.34
SAV0219	None	xylose isomerase	–1.58	–2.16
SAV0222	uhpT	antiporter [Staphylococcus sugar phosphate antiporter	–1.68	–2.16
SAV0259	scdA	Iron-sulfur cluster repair protein ScdA	–1.81	–2.05
SAV0261	lytR	LytR family transcriptional regulator	–1.91	–2.18
SAV0285	None	type VII secretion protein EsaB	–2.56	–2.51
SAV0315	nanA	N-acetylneuraminate lyase	–1.75	–2.25
SAV0320	geh	lipase	–2.81	–3.84
SAV0432	hsdS	restriction endonuclease subunit S	–1.98	–2.32
SAV0450	None	cobalamin synthesis protein CobW	–2.26	–2.82
SAV0458	None	sodium-dependent transporter	–1.31	–2.13
SAV0465	None	peptidase M23B	–3.07	–2.63
SAV0539	rplJ	50S ribosomal protein L10	–1.39	–2
SAV0631	None	manganese ABC transporter substrate-binding protein	–2.14	–2.16
SAV0632	None	membrane protein ABC transporter permease	–1.96	–2.14
SAV0633	None	phosphonate ABC transporter ATP-binding protein	–1.9	–2.09
SAV0705	saeS	histidine protein kinase	–2.9	–3.41
SAV0706	saeR	response regulator saeR	–1.82	–3.25
SAV0815	nuc	nuclease	–2.47	–3.07
SAV1052	truncated-atl	mannosyl-glycoprotein endo-beta-N-acetylglucosamidase	–2.72	–2.29
SAV1131	None	heme transporter IsdC	–1.58	–2.1
SAV1135	None	sortase B	–3.56	–2.71
SAV1136	None	heme-degrading monooxygenase IsdG, partial	–2.59	–3.96
SAV1155	None	fibrinogen-binding protein	–1.98	–3.14
SAV1158	None	fibrinogen-binding protein	–2.46	–2.85
SAV1159	None	fibrinogen-binding protein	–1.82	–2.12
SAV1163	None	alpha-hemolysin	–2.79	–4.02
SAV1169	argF	ornithine carbamoyltransferase	–1.85	–2.08
SAV1436	None	quinolone resistance protein NorB	–1.66	–2.05
SAV1437	None	amino acid permease	–1.62	–2.49
SAV1550	None	5-formyltetrahydrofolate cyclo-ligase	–2.09	–2.15
SAV1660	truncated-radC	hypothetical protein	–2.2	–2.6
SAV1661	None	type III leader peptidase	–1.84	–2.1
SAV1686	None	NrdR family transcriptional regulator	–1.79	–2.02
SAV1709	ald	alanine dehydrogenase	–1.72	–2.01
SAV1799	None	calcium-binding protein	–1.95	–2.27
SAV1809	splF	serine protease	–2.6	–4.04
SAV1810	splD	serine protease	–2.63	–4.02
SAV1811	splC	serine protease	–2.59	–3.87
SAV1812	splB	serine protease	–2.72	–4.2
SAV1813	splA	serine protease	–2.64	–4.18
SAV1819	lukD	gamma-hemolysin subunit B	–2.32	–3.44
SAV1820	lukE	gamma-hemolysin subunit A	–2.41	–3.05
SAV1909	None	cysteine protease	–2.21	–2.33
SAV1910	None	staphostatin A	–2.4	–2.05
SAV1914	None	Nitric-oxide synthase	–2.11	–2.18
SAV1937	None	extracellular adherence protein Eap/Map	–1.57	–2.48
SAV1938	None	protein map	–1.74	–2.24
SAV1942	None	inhibitor	–2.02	–3.32
SAV2004	None	gamma-hemolysin subunit B	–2.11	–3.37
SAV2005	None	succinyl-diaminopimelate desuccinylase	–1.64	–3.21
SAV2035	hld	delta-hemolysin	–1.87	–2.24
SAV2038	agrC	histidine kinase	–1.94	–2.09
SAV2039	agrA	histidine kinase	–2.09	–2.34
SAV2117	None	N5-glutamine S-adenosyl-L-methionine-dependent methyltransferase	–2.18	–2.11
SAV2119	tdk	thymidine kinase	–.94	–2.28
SAV2177	None	iron citrate ABC transporter substrate-binding protein	–2.09	–2.09
SAV2304	None	secretory antigen SsaA, partial	–3.37	–2.98
SAV2363	None	LytTR family transcriptional regulator	–1.94	–2.03
SAV2418	sbi	hypothetical protein	–2.04	–4.03
SAV2419	hlgA	gamma-hemolysin subunit A	–1.44	–3.81
SAV2420	hlgC	Gamma-hemolysin C subunit HlgC	–1.12	–3.55
SAV2421	hlgB	gamma-hemolysin subunit B	–1.24	–3.45
SAV2463	None	peptide ABC transporter ATP-binding protein	–2.21	–2.53
SAV2464	None	peptide ABC transporter ATP-binding protein	–2.68	–2.45
SAV2465	None	peptide ABC transporter permease	–1.99	–2.01
SAV2470	None	diaminopimelate epimerase	–1.55	–2.04
SAV2514	None	Probable transport protein	–2.05	–2.1
SAV2544	None	peptidase M23B	–2.89	–2.41
SAV2569	isaA	transglycosylase	–2.68	–2.39
SAV2632	arcC	carbamate kinase	–1.83	–2.18
SAV2634	arcB	ornithine carbamoyltransferase	–1.71	–2.15
SAV2662	None	capsular polysaccharide biosynthesis protein Cap8C	–2.9	–2.76
SAV2663	None	capsular polysaccharide biosynthesis protein Cap5B	–2.58	–2.69

The *in vitro* experiments showed that GA and NGA could effectively inhibit the growth, infection, adhesion, exotoxin secretion, and biofilm formation of MRSA, which was consistent with the key pathways identified in the GO and KEGG analyses.

#### Virulence Factors and Two-Component Systems

*S. aureus* is a pathogen that causes many diseases, including pneumonia, septicemia, and meningitis, which are caused by multiple virulence factors produced by this bacterium. These virulence factors are regulated by two-component systems (such as *agr*, *srrAB*, *arlRS*, *vraSR*, and *saeRS*) (Canovas et al., 2016).

The *saeR* and *saeS* genes were downregulated 3.53 and 4.32 times, respectively, after treatment with GA and 9.50 and 10.62 times, respectively, after treatment with NGA. Among the 16 TCS systems of *S. aureus*, *saeRS*play an important role in regulating more than 20 important virulence factors, such as hemolysins, leukocidins, coagulases and immune evasion molecules (Liu et al., 2016).

**Figure 6:**
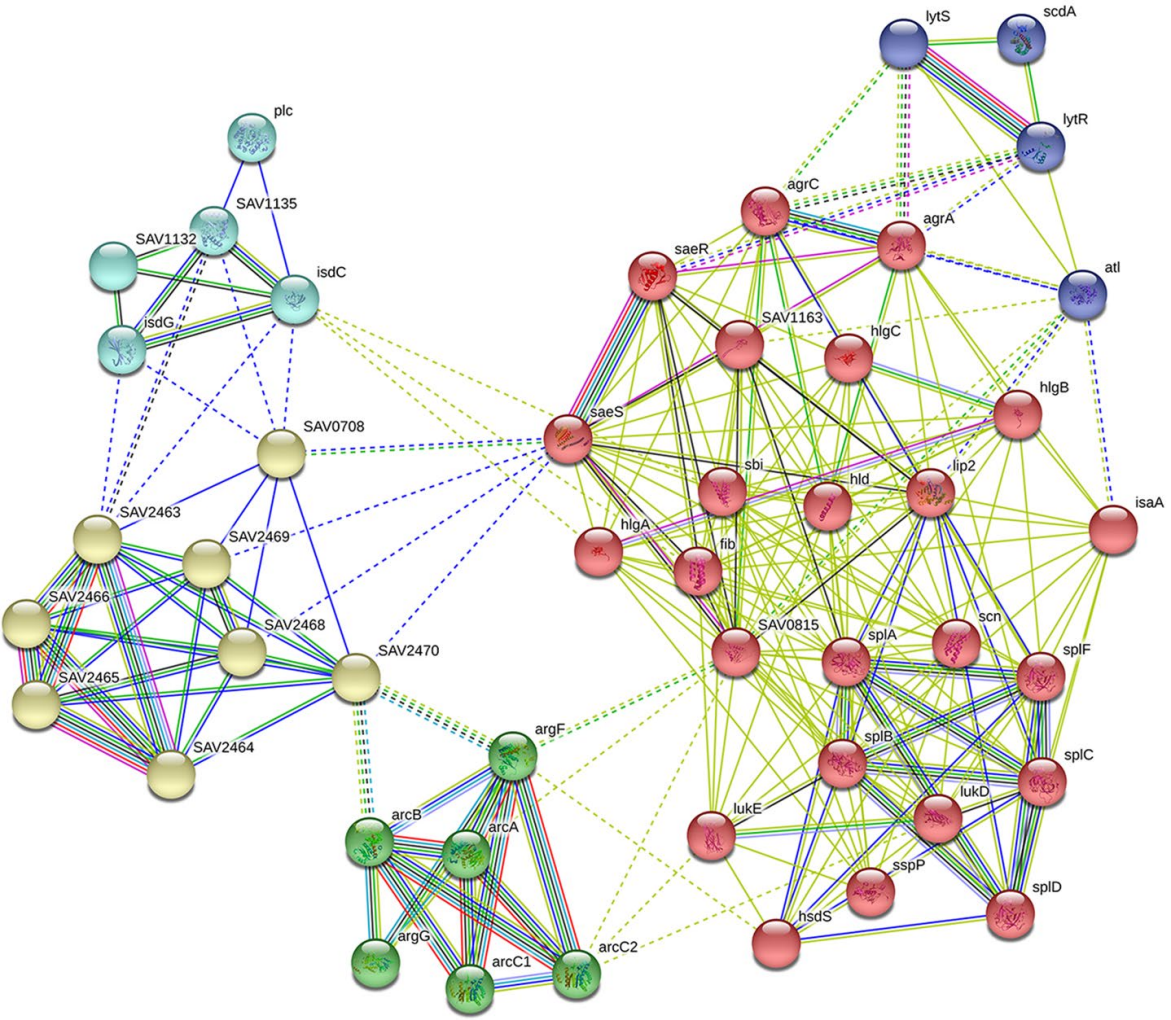
Gene interaction network including down-regulated expressed genes of *S. aureus* ATCC33591 cells treated with GA and NGA after using STRING bioinformatic tool.

 In our RNA-Seq results, we found that the expression levels of the following 16 genes associated with virulence factors were downregulated observably: the hemolysin-related genes *splABCDF* (*Spl* is involved in host colonization and infection and is considered to be a potential drug target) (Paharik et al., 2016); the IgG-binding protein related gene *sbi* (which can help bacteria escape macrophage phagocytosis and neutrophil killing) (Zhao et al., 2016); the delta-hemolysin gene *hld*; the gamma-hemolysin and leukocytotoxin-related genes *SAV2004*, *hlg*ABC and *Luk*DE; and three genes* SAV1155*, *SAV1158* and *SAV1159* which associated with fibrinogen-binding proteins. In addition, these virulence factors are capable of directly interacting with proteins in the *saeRS* two-component system. STRING network analysis was used to examine the relationships among the proteins whose expression decreased more than 4 times after GA and NGA treatment. A distinct network of *saeRS*-centric protein interactions was constructed and is shown in **Figure 6**. According to the results of the interaction analysis, *saeRS*-centered virulence factors and energy-metabolism-related proteins were significantly downregulated after treatment. Previous research has shown that the structural analogues of GA and NGA can inhibit *S. aureus* invasion of cells, the results of this study are consistent with our findings (Chaiyakunvat et al., 2016). By inhibiting the *saeRS* two-component system of MRSA strain, the expression of virulence factors of the strain was inhibited by the gambogic acid and neogambogic acid, thus inhibiting the invasion of MRSA strain to the host, which may be the main mechanism of GA and NGA antibacterial action.

In addition to virulence, both *agr* and *saeRS* influence biofilm formation in *S. aureus*, with *agr* acting *via* the production of phenol-soluble modulins (PSMs) (Surewaard et al., 2013) and *saeRS* by repressing the production of extracellular proteases that degrade proteins important for biofilm formation (Boles and Horswill, 2008).

Several compounds have been found to inhibit the expression of virulence-related genes in *S. aureus* by inhibiting the *agrAC* two-component system. Norlichexanthone has been shown to inhibit biofilm formation by inhibiting *agrAC* and *saeRS* expression. Some studies have shown that increased expression of the quorum sensing system can effectively inhibit biofilm formation (Baldry et al., 2016). Although *agrAC* and *saeRS* were inhibited after treatment with drugs such as GA, NGA, and norlichexanthone, the consequent downregulation of virulence factors may be the key to the inhibition of biofilm formation.

#### Cell Wall Formation

The significant direct inhibitory effects of GA and NGA on MRSA and the distinct shrinkage and rupture of the cell walls of the bacteria observed by SEM indicate that these compounds inhibit cell wall synthesis.

According to the transcriptomic analysis, expression of some of the key genes associated with cell wall formation was significantly inhibited, with inhibition ratios greater than 4. Capsular polysaccharides are important components of the cell wall. SAV2662 and SAV2663 are two capsular polysaccharide synthesis proteins that were significantly downregulated after treatment with GA and NGA.

In addition, after GA and NGA treatment, SAV0465 (N-acetylmuramoyl-L-alanine amidase), SAV0192 (N-acetylmuramic acid 6-phosphate etherase) and SAV0192 (phosphatase system sucrose-specific IIBC component) were significantly downregulated. Peptidoglycan forms an envelope structure in which bacteria maintain their morphology. This structure is formed by the crosslinking of N-acetylglucosamine (GlcNAc) and N-acetylmuramic acid (MurNAc) by short peptides. PGN encases the bacterial cell, forming a large, net-like, turgor-resisting and shape-maintaining envelope structure that is composed of glycan strands of two alternating β-1,4-linked sugars, GlcNAc and MurNAc, crosslinked by short peptides (Gutierrez et al., 2018).

MurNAc-6p is the product of MurNAc uptake and phosphorylation of MurNAc by the specific PTS transporter MurP, which plays an important role in the formation of peptide polysaccharides. SAV0191 and SAV0192 are the transcriptional regulators of MurNAc-6p in *S. aureus*. The results indicate that GA and NGA inhibit the cell wall formation in MRSA by inhibiting the synthesis of MurNAc, which could be an important drug target. In addition, *lip2* (a glycerol ester hydrolase) and SAV0631 (a lipoprotein) are two proteins related to cell wall formation that were also inhibited.

#### Q-RT-PCR and PRM

Based on RNA-Seq results, we hypothesized that GA and NGA could regulate virulence factors and other proteins directly associated with *saeRS* by inhibiting the expression of the *saeRS* proteins. To validate our hypothesis, Q-RT-PCR and PRM were conducted. Q-RT-PCR could accurately reflect the changes in MRSA gene expression before and after GA and NGA treatment. Due to the high specificity and sensitivity of PRM, this method has been widely used for the determination of target protein content.

**Figure 7:**
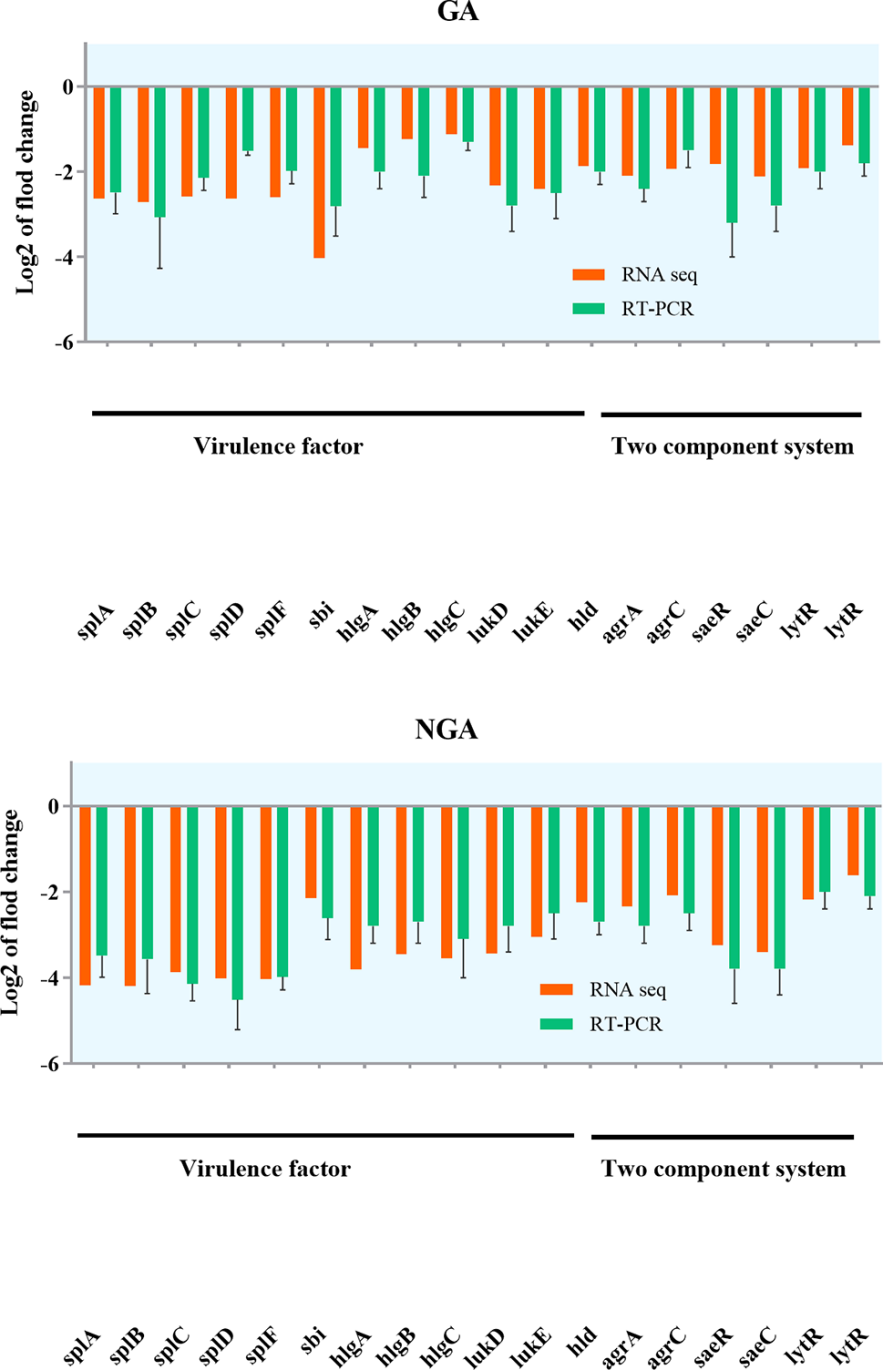
Validation of RNA-seq data for selected genes by real-time PCR.

Twenty differentially expressed proteins with distinct changes in expression were selected for Q-RT-PCR, and the detection results are shown in **Figure 7**. The variation trend for differential gene expression observed by Q-RT-PCR was consistent with that observed by RNA-Seq. Expression of 7 genes encoding the *saeRS*, *agrAC* and *sbi* proteins was detected before and after GA and NGA treatment by PRM. The results are shown in **Table 3**, we found that the expression of the proteins encoded by *saeRS* and *agrC* were get reduced after treatment with GA and NGA, which confirmed our hypothesis. However the differences in the expression of the other proteins were not significant.

**Table 3:** Quantity of protein expression based on the PRM detection.

Protein name	Gene name	Peptide sequence	Ration	
			GA vs. control	NGA vs. control
Response regulator SaeR	*saeR*	LDIPFIYLTAK	0.79	0.63
Histidine protein kinase SaeS	*saeS*	ILTNLLDNALK	0.86	0.61
Accessory gene regulator A	*agrA*	ELSQLDDR	0.81	1.27
Accessory gene regulator C	*agrC*	GLGLSTLK	0.82	0.65
Immunoglobulin-binding protein	*sbi*	GAIDQTVLTVLGSGSK	0.93	1.11
Gamma-hemolysin component C	*hlgC*	GSSDTSEFEITYGR	1.36	2.05
Delta-hemolysin	*hld*	WIIDTVNK	1.26	1.95

The results of this study suggested that both GA and NGA have significant anti-MRSA activity *in vivo* and *in vitro*, especially in the inhibition of biofilm formation and skin infection by MRSA. Transcriptome sequencing, RT-PCR and PRM were performed to elucidate the pathway *via* which GA and NGA downregulate the expression of the *saeRS*, a two-component system in MRSA, thus affecting the generation of virulence factors and biofilms by MRSA. In addition, GA and NGA also inhibited cell wall formation in the MRSA strains.

## Conclusion

This study reported the anti-MRSA activity of GA and NGA, including anti-biofilm formation activity *in vivo* and *in vitro*. GA and NGA were found to exert antibacterial activity by inhibiting the bacterial *saeRS* two-component system, providing new evidence for the development of anti-bacterial drugs. GA and NGA are cytotoxic but high sensitive and effective to MRSA, furthermore toxicity can be reduced by modification of the chemical structures of these compounds. Hence, we are confident that these compounds have potential applications as anti-MRSA drugs.

## Data Availability

All the sequencing reads have been submitted to the NCBI short-read archive (SRA) with accession number SAMN10230086.

## Ethics Statement

The animal experiment was approved by the Animal Ethics Committee of Harbin Veterinary Research Institute of the Chinese Academy of Agricultural Sciences.

## Author Contributions

XH and SL designed research. XH, YJ and QY analyzed data. XH, WZ, QY, ZD and YJ performed research. XH wrote the paper.

## Funding

The authors gratefully acknowledge the financial support from the Program for Natural Science Foundation of Heilongjiang Province of China (C2016063), the National Natural Science Funds of China (31602100), and the Central Public-Interest Scientific Institution Basal Fund (1610302017007).

## Conflict of Interest Statement

The authors declare that the research was conducted in the absence of any commercial or financial relationships that could be construed as a potential conflict of interest.

## Abbreviations

MRSA, *Staphylococcus aureus*; GA, Gambogic acid; NGA, Neogambogic acid; TCS, Two-component signaling; MIC, Minimal inhibitory concentration; MBC, Minimum bactericidal concentration; PVL, Panton-Valentine leukocidin; PRM, Parallel reaction monitoring; SEM, Scanning electron microscopy; GlcNAc, N-acetylglucosamine; MurNAc, N-acetylmuramic acid.
