# Supplementary material for: Transcriptional Analysis of the Effects of Gambogic Acid and Neogambogic Acid on Methicillin-Resistant Staphylococcus aureus
Source: Front Pharmacol. 2019 Sep 13;10:986. doi: 10.3389/fphar.2019.00986 (PMC6753875; doi:10.3389/fphar.2019.00986)
Supplement: Supplementary file 1 [file Table_1.docx]

Table S1. Primers used in this study.

| **Gene** | **Primer direction** | **Primer sequence (5’→3’)** |
| --- | --- | --- |
| *splA* | Sense | GTAGCAGCACATTATTCG |
|  | Antisense | TTCTATCTTTCGCTTTTG |
| *splB* | Sense | GTGATAAAGGTAATGGTGGTA |
|  | Antisense | TAAAAGCAACTACACC |
| *splC* | Sense | ATTCAGGATCACCAGT |
|  | Antisense | TTGAGGCGTAAAGTAT |
| *splD* | Sense | TAACGGTGGTGGACTT |
|  | Antisense | ATTTTCTACCTTTTGG |
| *splF* | Sense | AATAACGGTGGTGGACT |
|  | Antisense | ATCTTTGAATTTTCTACCTTTTGGT |
| *Sbi* | Sense | AGCAATCGTTCGTCAT |
|  | Antisense | TTTCTTTTCAGCATCT |
| *hlgA* | Sense | ACACCAGTCAGCGTAA |
|  | Antisense | CACAAAGCCTGAGTTA |
| *hlgB* | Sense | GGGGAGCTAAATACAAT |
|  | Antisense | GTCCACCAGATAAACCA |
| *hlgC* | Sense | TATCGCCACAGTATCTC |
|  | Antisense | ACTCTATGTCCGTCTAA |
| *lukD* | Sense | GTTTCGGTTAGTTCAG |
|  | Antisense | ATCCATTCAATCCACC |
| *lukE* | Sense | GCCTTTAGCATCTCCG |
|  | Antisense | TTACTACTTACATCCTCC |
| *hld* | Sense | TTCAATGGCACAAGAT |
|  | Antisense | TTTTACTAAGTCACCG |
| *agrA* | Sense | TTCATTTGCGAAGACG |
|  | Antisense | TTATCAGTTGCGAGGG |
| *agrC* | Sense | TGATCCTGCAATACTC |
|  | Antisense | GCAAATAATACGAATG |
| *saeR* | Sense | TTAACTGCCAAAACAC |
|  | Antisense | TTAACTGCCAAAACAC |
| *saeS* | Sense | AAACAAGCAAACAGAT |
|  | Antisense | TTCGCTACAGAAATTCAC |
| *lytR* | Sense | TGTAAAAGAAACATTGGAAG |
|  | Antisense | AATAATCGCAGGTGGC |
| *lytS* | Sense | GCTATCGCTAACAATG |
|  | Antisense | AAGGCAAACAAACTGAA |
